# N-glycosylation of Viral E Protein Is the Determinant for Vector Midgut Invasion by Flaviviruses

**DOI:** 10.1128/mBio.00046-18

**Published:** 2018-02-20

**Authors:** Dan Wen, Suhua Li, Fangfang Dong, Yanan Zhang, Yongfang Lin, Jumei Wang, Zhen Zou, Aihua Zheng

**Affiliations:** a State Key Laboratory of Integrated Management of Pest Insects and Rodents, Institute of Zoology, Chinese Academy of Sciences, Beijing, China; b University of Chinese Academy of Sciences, Beijing, China; Mailman School of Public Health, Columbia University

**Keywords:** flavivirus, glycosylation, mosquito, transmission, Zika

## Abstract

Transmission of flaviviruses by hematophagous insects such as mosquitoes requires acquisition of the virus during blood feeding on the host, with midgut as the primary infection site. Here, we report that N-glycosylation of the E protein, which is conserved among most flaviviruses, is critical for the Zika virus (ZIKV) to invade the vector midgut by inhibiting the reactive oxygen species (ROS) pathway of the mosquito immune system. Our data further show that removal of the ZIKV E glycosylation site prevents mosquito infection by flaviviruses via the oral route, whereas there is no effect on infection by intrathoracic microinjection, which bypasses the midgut. Interestingly, the defect in infection of the mosquito midgut by the mutant virus through blood feeding is rescued by reduction of the ROS level by application of vitamin C, a well-known antioxidant. Therefore, our data demonstrate that ZIKV utilizes the glycosylation on the envelope to antagonize the vector immune defense during infection.

## IMPORTANCE

Most flaviviruses, including Zika virus (ZIKV), are transmitted between hosts by arthropod vectors, such as mosquitoes, which acquire the virus during a blood meal. Here, by mutagenesis, we found a major role of the N-glycosylation of flavivirus E protein in its transmission circle, facilitating its survival against the vector immune system during invasion of the mosquito midgut while blood feeding on the host. In spite of the extensive studies of the involvement of N-glycan modification of flavivirus E protein in virus-host interactions, we discovered its critical role in virus-vector interaction and the evolution of flavivirus. Given the deleterious effects of ZIKV on human health, this study might have a significant impact on development of novel transmission-blocking strategies.

## INTRODUCTION

The genus *Flavivirus*, belonging to the family *Flaviviridae*, includes many highly pathogenic viruses, such as Zika virus (ZIKV), dengue virus (DENV), Japanese encephalitis virus (JEV), West Nile virus (WNV), yellow fever virus (YFV), Saint Louis encephalitis virus (SLEV), Murray Valley encephalitis virus (MVEV), and tick-borne encephalitis virus (TBEV). The members of this highly pathogenic group *Flavivirus* are mostly transmitted by mosquitoes and ticks. Recently, mosquito-borne flaviviruses, such as ZIKV, DENV, and WNV, have been spreading worldwide, causing hundreds of millions of infections annually (1, 2). In spite of the huge public health burden, effective licensed vaccines and antiviral therapies are still under development for most flaviviruses (3, 4).

ZIKV was first isolated in Uganda in 1947 (prototype strain MR766) (5, 6) and was endemic in the tropical areas of Africa and Asia for a long time, with the yellow fever mosquito *Aedes aegypti* as its primary vector (7). Since 2007, there have been a series of outbreaks on various islands in the Pacific Ocean, including Yap Island and French Micronesia (8). The first local case in the Americas was reported in northeast Brazil in 2015 (9), followed by a pandemic spreading from South America to North America (10, 11). Apart from Zika fever, ZIKV infections cause other severe diseases, including Guillain-Barré syndrome and testis damage (12–15). Zika virus could also be passed from a pregnant woman to her fetus, resulting in defects in babies, such as microcephaly. Phylogenetic analysis suggests that ZIKV consists of African and Asian lineages. While MR766 belongs to the African lineage, the Pacific Ocean and American strains belong to the Asian lineage (16, 17).

ZIKV is a spherical, single-stranded, positive RNA virus enveloped by a lipid bilayer, which is covered by M and E glycoproteins. The E protein forms an elongated structure composed of three β-strand-rich domains with a transmembrane domain and a hydrophobic fusion loop on opposite sides (18–20). The E proteins are responsible for receptor binding and membrane fusion (21–25) and are also the major targets of neutralizing antibodies (26). Usually, there are up to two N-glycosylation sites on the E protein of vector-borne pathogenic flaviviruses, except for YFV (27). One highly conserved glycosylation site localizes at N^153^ or N^154^ (depending on the particular virus) of E protein, while another one at N^67^ is only found on the dengue virus (28–30). ZIKV E protein is N-glycosylated at N^154^, which is located on a loop close to the fusion peptide of a neighboring E protein (21, 27, 31, 32).

Glycosylation of the asparagine (N) residue within the conserved N-glycosylation motif N-X-(S/T) is the most common posttranslational modification on viral envelope, implying important functions in viral growth and pathogenesis (33). In contrast to the conserved E glycosylation in the clinical isolates, extensive passages of laboratory isolates of flaviviruses, such as DENV, ZIKV, and WNV, in tissues and cell cultures can sometimes lead to the deletion of E N^153/154^-glycosylation modifications (16, 34, 35). These evidences suggest that the E N^153/154^ glycosylation on the envelope is not critically important for the growth of flaviviruses *in vitro*. Mutagenesis studies on many flaviviruses, including DENV, WNV, and ZIKV, indicate that the loss of N^153/154^-glycosylation modifications can reduce the viral growth in mammalian and mosquito cell cultures by various levels, but cannot block it completely (28, 36–42). Studies on the vector competence of WNV suggest that the E N^153^-glycosylation is required for viral growth in *Culex pipiens* and *Culex tarsalis* (36), but not *C. pipiens pallens* (43) and *Culex quinquefasciatus* (44). However, E N^153^-glycosylation-negative DENV inoculated into *A. aegypti* by intrathoracic injection shows a similar replication level to the wild type (WT) (37). A recent mutagenesis study on ZIKV E N^154^-glycosylation shows diminished virus infection of *A. aegypti* mosquitoes by oral route (45). Taken together, the selection pressure upon the N^153/154^-glycosylation of E protein during the flavivirus transmission cycle is still unclear.

Insects depend on their innate immune system to combat invading viruses and parasites (46). Hematophagous mosquitoes need blood nutrition for egg maturation, which makes them vectors of devastating infectious diseases (47, 48). After a blood meal, the midgut epithelia become the first line for contacting pathogens (49). The immune deficiency (IMD) pathway and reactive oxygen species (ROS) are implicated in mucosal immunity and are involved in elimination of invading microbes (50, 51). It is reported that *Wolbachia* increases the ROS level of a mosquito to defend against DENV infection (50). However, a high ROS level could also damage host cells and tissues. Removal of excessive ROS depends on antioxidants, such as superoxide dismutases (SODs), catalases, and hypoxanthines (HPXs) (52).

In this study, we addressed the role of N-glycosylation of ZIKV E protein during viral infection of *A. aegypti*, which is an essential step for viral transmission. By site-directed mutagenesis, we found that the E N^154^-glycosylation is critical for antagonizing the mosquito immune system during invasion of the midgut. Additionally, a ZIKV strain with mutation for E N^154^-glycosylation could not infect the midgut of *Aedes* mosquito, and this phenotype could be rescued by vitamin C (VC), an inhibitor of the ROS pathway. Our data therefore identify E N^154^ as a determinant of ZIKV transmission via mosquitoes. Given the deleterious effects of ZIKV on human health, findings from this study might have a significant impact on development of novel transmission-blocking strategies against flaviviruses.

## RESULTS

### Removal of the N-glycosylation site of E protein does not affect ZIKV infectivity in the mammalian cell culture system.

**FIG 1:**
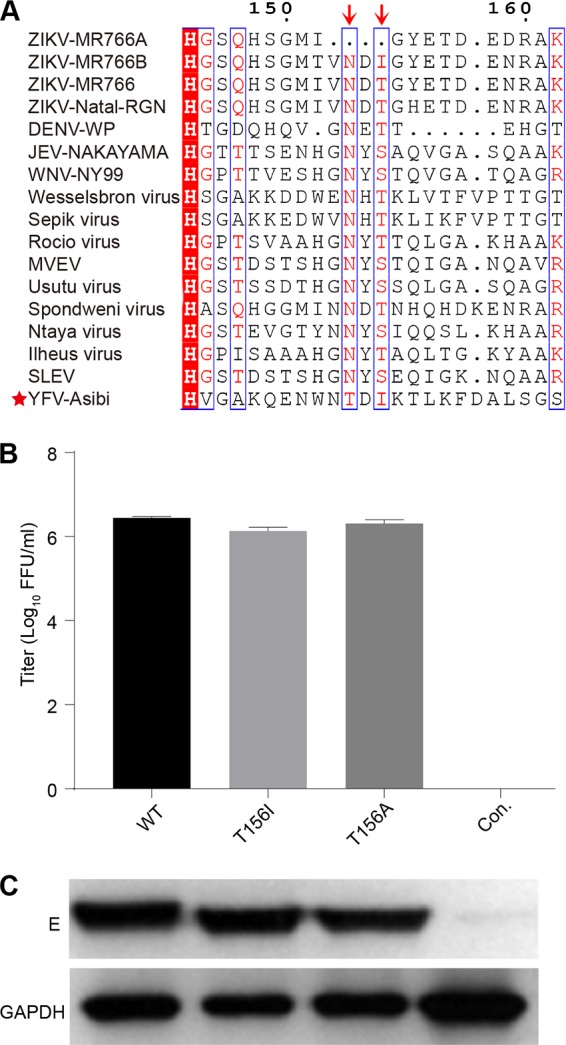
Removal of the N-glycosylation site of the E protein does not affect ZIKV infectivity. (A) Sequence comparison of the E protein N-glycosylation region (E144–162, numbered according to the ZIKV MR766a isolate) of representative flaviviruses. Shown are sequences for ZIKV MR766a with a 4-amino-acid (aa) deletion at the N-glycosylation site (GenBank accession no. AY632535), ZIKV MR766b with a T156I mutation in the E protein (LC002520), ZIKV MR766 with intact N^154^-glycosylation (HQ234498), ZIKV Natal-RGN (KU527068), dengue virus western Pacific strain (DENV WP; U88535), Japanese encephalitis virus NAKAYAMA strain (JEV NAKAYAMA; EF571853), West Nile virus NY99 strain (WNV NY99; DQ211652), Wesselsbron virus (NC_012735), Sepik virus (NC_008719), Rocio virus (AY632542), Murray Valley encephalitis virus (MVEV; NC_000943), Usutu virus (NC_006551), Spondweni virus (NC_029055), Ntaya virus (NC_018705), Ilhéus virus (NC_009028), Saint Louis encephalitis virus (SLEV; NC_007580), and yellow fever virus Asibi strain (YFV Asibi; AY640589). The sequence alignment was generated by ClustalW software. Yellow fever virus (YFV), with no E N-glycosylation, is highlighted with a star. The arrows indicate the first and third amino acids of the N-X-(S/T) glycosylation motif. (B) RVPs were produced in HEK293T cells by cotransfection of the plasmid encoding the structural protein of ZIKV MR766 strain and the WNV replicon plasmid with a GFP reporter. The titers of RVPs in the supernatant were determined 72 h posttransfection, and infection efficiency was gauged on Huh7.5 cells. Both wild-type (WT) and mutant virus (T165I and T156A) RVPs showed similar titers, while zero titer was detected in the control (Con.) transfected only with the WNV replicon plasmid (*n* = 3). Error bars indicate standard deviation (SD). (C) E protein in HEK293T cell lysates was blotted by an anti-E monoclonal antibody 4G2 (E) using anti-glyceraldehyde-3-phosphate dehydrogenase (GAPDH) as a control. Each lane represents protein lysate of the transfected cells from which the corresponding RVPs listed above were produced. The results are representative of three independent experiments.

Given the fact that the N^153/154^-glycosylation modification of E protein of flaviviruses is easy to lose during passages in cell cultures system due to mutation or deletion in the N-X-(S/T) motif (16, 34, 35) (Fig. 1A), we hypothesize that the E N-glycosylation is not necessary for ZIKV infection *in vitro*. To test this hypothesis, we first developed a ZIKV recombinant virus particle (RVP) system that supports only single-cycle infection, thus making it the ideal candidate to test virus infectivity (53, 54). Human codon-optimized prME (CprME) of ZIKV prototype MR766 strain with an intact E N^154^-glycosylation site (GenBank accession no. HQ234498) was cloned into the pCDNA3.1 plasmid, and the ZIKV RVPs were produced by cotransfection of this plasmid along with a WNV replicon plasmid encoding a green fluorescent protein (GFP) reporter into HEK293T cells. Infectious ZIKV RVPs were efficiently produced at a titer of ~2.6 × 10^6^ focus-forming units (FFU)/ml as determined on Huh7.5 cells (Fig. 1B) (53, 54). To test the effect of N-glycosylation on viral infectivity, the T in the third position of the N^154^-D-T^156^ motif was substituted for with either A (T156A) or I (T156I) to remove the N-glycosylation site, without introducing major changes in the side chain. As shown in Fig. 1C, the E-T156A and E-T156I proteins migrated faster than the WT, due to the loss of the glycosylation site. However, as evidenced from the band intensity, the incorporated mutations did not affect the expression level of the protein. The titer of WT RVP was 2.6 × 10^6^ FFU/ml, while those of the T156A and T156I RVPs were 1.4 × 10^6^ and 2.1 × 10^6^ FFU/ml, respectively (Fig. 1B). These results suggest that the ZIKV E glycosylation has minimal effect on viral infectivity *in vitro*.

### Removal of the N-glycosylation site of E protein does not affect ZIKV replication *in vitro.*

**FIG 2:**
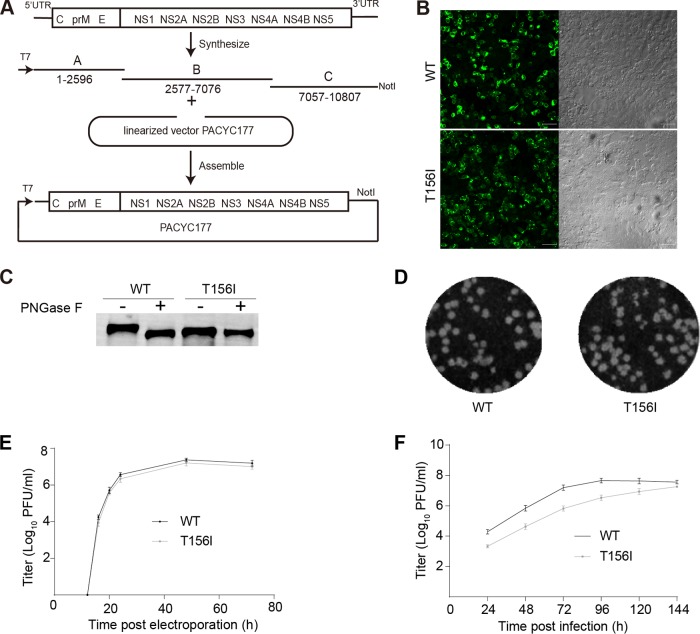
Removal of E N-glycosylation does not affect ZIKV replication in mammalian cell culture. (A) Schematic diagram of the ZIKV genome organization and the construction of the MR766 infectious clone. Three cDNA fragments designated A to C were synthesized and assembled into a low-copy-number PACYC177 vector with a T7 promoter at the 5′ end and a NotI linearization site at the 3′ end. The viruses were rescued by transfection of the *in vitro*-transcribed RNA into HEK293T cells. (B) E protein expression in both wild-type (WT) and mutant virus (T156I) infectious clone-transfected cells was detected by immunofluorescence using anti-E 4G2 antibody (left). The right column is the bright-field image. The size bar indicates 50 μm. (C) Endoglycosidase analyses of E protein. WT and E-T156I viral particles were pelleted and treated with PNGase F overnight at room temperature. Enzyme-digested samples were blotted by anti-E monoclonal antibody 4G2. This is a representative example from three independent experiments. (D) Both the WT and E-T156I mutant virus showed similar plaque-forming efficiencies on Vero cells. (E) Virus samples in the supernatant of transfected HEK293T cells were collected at different time points, and the titers were tested by plaque assay on Vero cells. The titer of the ZIKV MR766 WT reached 6.8 × 10^6^ PFU/ml at 24 h postelectroporation and peaked at 7.9 × 10^7^ PFU/ml at 48 h (*n* = 3). Error bars indicate SD. This is a representative example from three independent experiments. Both the WT virus and E-T156I mutant virus exhibited similar titers at all time points. (F) Multistep growth curve of WT and E-T156I mutant ZIKV MR766. C6/36 cells were infected by the WT and mutant viruses at an MOI of 0.01. Virus samples from the supernatant were collected at different time points and gauged by a plaque assay on Vero cells (*n* = 3). Error bars indicate SD. The results are representative of three independent experiments.

To test the effect of E protein N-glycosylation on ZIKV replication, an infectious clone of ZIKV prototype MR766 was produced by *de novo* synthesis. There are various ZIKV MR766 sequences available in GenBank, obtained from isolates with different passage histories (16). GenBank accession no. LC002520 is the only sequence one that includes the full 5′- and 3′-untranslated regions when we designed the experiment, but it lacks the glycosylation site because of the E-T156I mutation. On the other hand, HQ234498 lacks the full 5′ and 3′ untranslated regions, but the EN^154^ glycosylation site is intact. So, we synthesized the ZIKV MR766 mostly based on LC002520, except the EN^154^ glycosylation region which was designed based on HQ234498. The resulting sequence is almost the same as that of the recently reported MR766 infectious clone by Matthew J. Evans’s lab derived from a viral isolate (55) (GenBank accession no. KX830960), aside from 3 nonsense mutations at nucleotides 1669, 4519, and 5512. Briefly, the MR766 sequence was synthesized and assembled into the pACYC177 vector with a T7 promoter upstream of the first nucleotide of viral genome and a NotI restriction site following the 3′-terminal end (Fig. 2A) (56, 57). The infectious clone was stable for at least 5 generations during its propagation in TOP10 cells.

ZIKV MR766 viruses were rescued by electroporation of the *in vitro*-transcribed RNA into HEK293T cells. Viral protein expression was detected after electroporation using an antibody against the E protein (Fig. 2B). The rescued ZIKV WT virus formed plaques on Vero cells (Fig. 2D). After electroporation of the HEK293T cells, the titer of the ZIKV MR766 WT reached 6.8 × 10^6^ PFU/ml at 24 h postelectroporation and peaked at 7.9 × 10^7^ PFU/ml at 48 h postelectroporation, as gauged on Vero cells by a plaque assay (Fig. 2E).

We then tested the effect of E N-glycosylation on viral replication by introducing an E-T156I mutation into the ZIKV MR766 infectious clone by site-directed mutagenesis. The electroporated HEK293T cells showed similar levels of expression for both WT and mutant E proteins (Fig. 2B). To confirm the loss of E N^154^-glycosylation, the viral particles in the supernatant were pelleted and treated with peptide-*N*-glycosidase F (PNGase F), and the mutant E protein migrated at the same position as that of the endoglycosidase-digested WT viruses (Fig. 2C). The plaque sizes produced by the WT had a diameter 1.2 times of those of the mutant, as measured by ImageJ (Fig. 2D). As shown in Fig. 2E, the titer of glycosylation-negative viruses in the supernatant is only about 0.4-log fold lower than that of the WT.

We further analyzed the effect of N-glycosylation on viral replication in mosquito cell cultures by a multistep growth curve assay. Mosquito C6/36 cells were infected at a multiplicity of infection (MOI) of 0.01 with WT or mutant MR766 virus followed by viral titer measurements in the supernatant, performed by plaque assays on Vero cells. Although the mutant showed a slower early growth rate, the peak titers of the WT and glycosylation-negative mutants were similar by 7 days postinfection (Fig. 2F). These results suggest that the E N-glycosylation has minimal effect on ZIKV replication in both mammalian and insect cell cultures.

### E protein N-glycosylation-negative ZIKV fails to cross the midgut barrier of *A. aegypti* mosquitoes.

**FIG 3:**
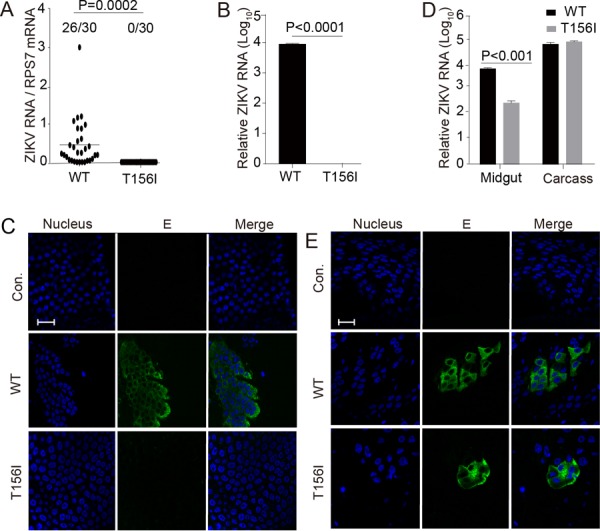
N-glycosylation site mutation of E protein blocks vector midgut invasion by ZIKV. (A and B) Five- to 6-day-old female *A. aegypti* mosquitoes were blood fed along with wild-type (WT) or mutant (T156I) ZIKV diluted to 1.5 × 10^5^ PFU/ml. (A) Seven days postinfection, total RNA from the whole body of a single mosquito was extracted, and the viral RNA level was tested by real-time PCR. The ZIKV viral RNA level was normalized against the reference gene ribosomal protein gene S7 (RPS7). Thirty mosquitoes were tested from each group; the number of infected mosquitoes is shown on the left. Twenty-six mosquitoes were infected by WT virus, whereas no mosquito was infected by mutant virus. (B) Ten mosquitoes were pooled for virus detection by real-time PCR, as mentioned above. Viral RNA could be detected in the WT virus-infected mosquitoes, whereas no viral RNA was found in the mutant (T156I)-infected mosquitoes. (C) Seven days postinfection by blood meal, midguts of mosquitoes treated as in panel A were dissected and viral infections were detected using anti-E 4G2 antibody (E) by immunofluorescence assay. Nuclei were stained by Hoechst 33342 (Nucleus). The bar indicates 20 μm. Viral protein E could be detected in WT virus-infected mosquito midgut compared to its absence in mutant virus-infected mosquitoes. (D and E) Five- to 6-day-old female *A. aegypti* mosquitoes were inoculated with 60 PFU of WT or E-T156I ZIKV by intrathoracic injection. (D) Total RNA samples from 20 midguts or the carcass (mosquito body without the midgut) were extracted separately 7 days postinfection, and the viral RNA levels were detected by real-time PCR as in panel B. The carcass of the mosquito shows similar levels of WT and mutant virus (T156I) infection (right panel). Additionally, in contrast to oral infection, mutant virus (T156I) is detected in the mosquito midgut when injected intrathoracically, although at a level lower than the WT (left panel). (E) Viral infection in the midgut was also detected 7 days postinfection by immunofluorescence assay as in panel C. Viral E protein was detected in both WT- and mutant virus-infected mosquito midguts when injected intrathoracically. The bar indicates 20 μm. Panels A to E are representatives of more than five independent experiments. The *P* value was determined by unpaired *t* test.

As expected, the E N-glycosylation mutation did not significantly affect ZIKV infection and replication *in vitro*. Next, we analyzed its effect on in-vector competence by determining the impact of E N^154^-glycosylation on ZIKV infection of *A. aegypti* mosquitoes. A blood meal containing 1.5 × 10^5^ PFU/ml of either WT or glycosylation-negative viruses diluted in 50% Dulbecco’s modified Eagle’s medium (DMEM) and 50% mouse blood was fed to the laboratory-reared UGAL/Rockefeller strain of *A. aegypti*. After 7 days, total RNA was extracted from the whole mosquito, and ZIKV viral RNA was analyzed by real-time PCR. The ZIKV RNA levels were normalized against reference gene ribosomal protein gene S7 (RPS7). The WT ZIKV could infect the mosquitoes efficiently, with nearly 87% of the total mosquitoes infected, while none of the mosquitoes were infected by the glycosylation-deficient mutant viruses (Fig. 3A). Ten mosquitoes from each group were pooled, and total RNA was extracted for measurement of ZIKV RNA by real-time PCR. (Fig. S2A in the supplemental material shows the standard curve.) Supporting our results presented above, mosquitoes were infected efficiently by the WT virus, whereas almost no infection could be detected in the case of the mutant (Fig. 3B). The region encoding the prME protein was sequenced, and no reversion of the glycosylation mutation was detected (see Fig. S1 in the supplemental material). The midguts were dissected to test for ZIKV infection by immunofluorescence, utilizing an antibody targeting the viral E protein. Midguts from mosquitoes treated with WT virus were found to be mostly positive for ZIKV infection, while the ones treated with the mutants were not (Fig. 3C).

10.1128/mBio.00046-18.1 FIG S1  Sequence of E-T156I MR766 virus in mosquitoes 7 days postinfection. The region encoding the prME region was sequenced, and no revertant mutation was detected. Underlined ATA encodes E-I156. Download FIG S1, TIF file, 0.1 MB. Copyright © 2018 Wen et al. 2018 Wen et al. This content is distributed under the terms of the Creative Commons Attribution 4.0 International license.

To identify the bottleneck that is affecting the virulence of the glycosylation-deficient mutants, *A. aegypti* mosquitoes were injected intrathoracically with 60 PFU of WT or mutant viruses to bypass the midgut barrier. Seven days postinfection, the midgut and the carcass were separated for virus detection by real-time PCR. Both the WT and mutant could successfully infect the mosquito carcass to an equal extent. Surprisingly, in contrast to the oral route, the mutant ZIKV could penetrate the epithelia of midgut by the thoracic route, although the RNA level of mutant virus was about 1 log-fold lower than that of the WT (Fig. 3D). The infection of midguts was confirmed by immunofluorescence with an anti-E protein antibody. Our results revealed successful infection by both the WT and mutant, although the WT was found to be more efficient than the mutant (Fig. 3E). These results suggest that the N-glycosylation on the E protein is critical for ZIKV invasion of the mosquito midgut by the oral route.

**FIG 4:**
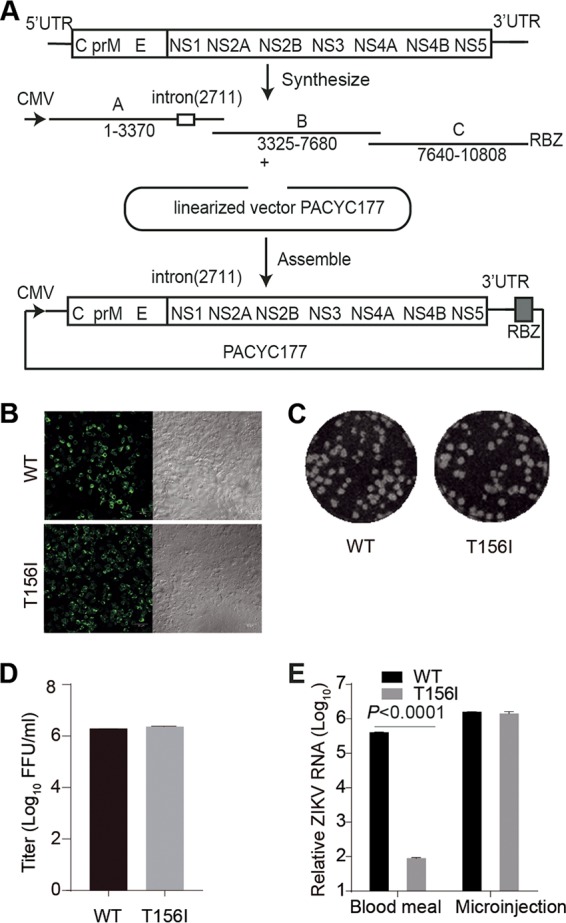
E-T156I mutation inhibits the midgut invasion of the Natal-RGN strain of ZIKV. (A) Schematic diagram of construction of the Natal-RGN infectious clone. Three cDNA fragments designated A to C were synthesized and assembled in the PACYC177 vector under the CMV promoter. An intron was inserted after nucleotide 2711 of the viral sequence, and an HDV ribozyme (RBZ) was inserted downstream from the 3′ end of the ZIKV genome. An E-T156I mutation was introduced to delete the E N^154^-glycosylation site. The viruses were rescued by transfection of the infectious clone plasmid into HEK293T cells. The viral RNA was sequenced, and the intron was completely spliced. (B) The E protein expression in the cells was detected by immunofluorescence with anti-E 4G2 antibody (left). The right column is the bright-field image. The bar indicates 50 μm. (C) Both the wild-type (WT) and E-T156I mutant (T156I) ZIKV virus formed plaques on Vero cells. (D) WT and E-T156I viruses were collected 4 days posttransfection and showed similar titers, as measured by plaque assay on Vero cells (*n* = 3). Error bars indicate SD. (E) Five- to 6-day-old female *A. aegypti* mosquitoes were fed a blood meal containing the WT or mutant (T156I) ZIKV Natal-RGN strain diluted to 5 × 10^5^ FFU/ml or infected with 60 FFU of WT or E-T156I ZIKV by intrathoracic injection (microinjection). Seven days postinfection, total RNA samples from 10 mosquitoes were extracted, and the viral RNA levels were detected by real-time PCR (*n* = 3). Error bars indicate SD. The *P* value was determined by unpaired *t* test. Panels B to E are representatives of three independent experiments.

Besides the African lineage strain MR766, we also tested the effect of E N-glycosylation on vector competence using a ZIKV Asian strain, Natal-RGN. Natal-RGN is representative of the strain endemic to Brazil since 2015. The complete genome sequence of Natal-RGN (GenBank accession no. KU527068) was recovered directly from the fetal brain tissue of a pregnant patient infected in Brazil in 2015 (58). We developed WT and E glycosylation-negative (E-T156I) infectious clones of the Natal-RGN strain by *de novo* synthesis and cloning into the PACYC177 vector containing a mammalian cytomegalovirus (CMV) promoter (Fig. 4A). The viruses were rescued successfully by transfection of the infectious clone plasmids directly into the HEK293T cells. Both WT and mutant E protein were expressed efficiently in the transfected HEK293T cells (Fig. 4B), and the virus titers in the supernatant were found to be similar (Fig. 4D), as gauged by a plaque assay (Fig. 4C). Consistent with the results from MR766, the Natal-RGN E glycosylation negative mutant infected *A. aegypti* mosquitoes at a level similar to that of the WT infections when administered by intrathoracic microinjection. However, infection by the mutant virus administered through blood feeding decreased by about 3.5-log fold compared to the WT, as assayed by viral RNA levels in the whole mosquito using real-time PCR (Fig. 4E). (Fig. S2A shows the standard curve.) The region encoding prME was sequenced, and no revertant mutations were detected (data not shown).

10.1128/mBio.00046-18.2 FIG S2  Standard curve for the real-time PCR assays for the detection of ZIKV MR766 (A) and Natal-RGN (B). The sizes of the PCR products are 141 bp and 122 bp for MR766 and Natal-RGN, respectively. The correlation coefficients (*R*^2^) were 0.9970 and 0.9990 for MR766 (A) and Natal-RGN (B), respectively. The linear dynamic ranges of both assays were higher than 6 log fold, and all the threshold cycle (Ct) values in the following experiments fell into the dynamic ranges. The results are representative of two independent experiments. Download FIG S2, TIF file, 2.8 MB. Copyright © 2018 Wen et al. 2018 Wen et al. This content is distributed under the terms of the Creative Commons Attribution 4.0 International license.

It is still plausible that the failure of midgut evasion was caused by mutation of the amino acid sequence rather than the change of glycosylation. To exclude this possibility, we further introduced an E-N154Q mutation, which is a common mutation to abolish N-glycosylation (45), into the MR766 infectious clone. As expected, the E-N154Q mutation decreased the virus infection through the oral route by 3.5 log compared with the WT, while no obvious change was detected by the intrathoracic route (see Fig. S3D in the supplemental material). No obvious changes were detected on the viral protein expression (Fig. S3A), plaque formation (Fig. S3B), and viral titers (Fig. S3C) of the mutant virus.

10.1128/mBio.00046-18.3 FIG S3  E-N154Q mutation inhibits the midgut invasion of the ZIKV MR766 strain. (A) An E-N154Q mutation was introduced into the MR766 infectious clone to remove the E N^154^-glycosylation modification. The viruses were rescued by transfection of the infectious clone plasmid into HEK293T cells. The E protein expression in the cells was detected by immunofluorescence with anti-E 4G2 antibody (left). The right column is a bright-field image. The bar indicates 50 μm. (B) Both the wild-type (WT) and E-N154Q mutant (N154Q) ZIKV virus formed plaques on Vero cells. (C) WT and E-N154Q viruses were collected 6 days posttransfection and showed similar titers, as measured by plaque assay on Vero cells (*n* = 3). The error bar indicates SD. (D) Five- to 6-day-old female *A. aegypti* mosquitoes were fed a blood meal containing the WT or mutant (N154Q) ZIKV MR766 strain diluted to 1.5 × 10^5^ FFU/ml or infected with 60 FFU of WT or E-N154Q ZIKV by intrathoracic injection (Microinjection). Seven days postinfection, total RNA from 10 mosquitoes was extracted, and the viral RNA levels were detected by real-time PCR (*n* = 3). Error bars indicate SD. The above data are representative of three independent experiments. Download FIG S3, TIF file, 0.9 MB. Copyright © 2018 Wen et al. 2018 Wen et al. This content is distributed under the terms of the Creative Commons Attribution 4.0 International license.

### Vitamin C can rescue midgut invasion of mutant ZIKV by decreasing the ROS level.

**FIG 5:**
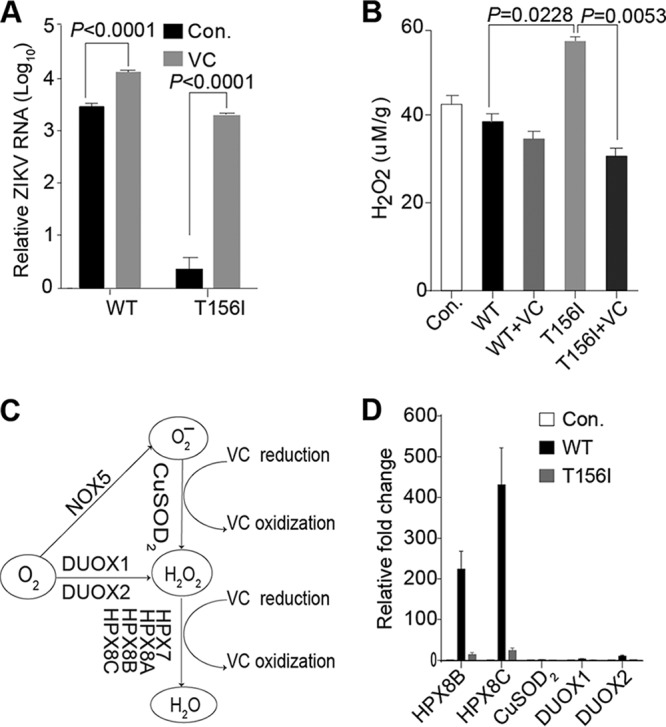
Inhibition of ROS production by vitamin C (VC) can rescue mutant ZIKV infection of mosquito midgut by oral infection. (A) Five- to 6-day-old female *A. aegypti* mosquitoes were blood fed along with wild-type (WT) or mutant (T156I) ZIKV supplemented with 0 or 20 mM VC. Seven days postinfection, total RNA pooled from 10 mosquitoes was extracted, and ZIKV viral RNA levels were tested by real-time PCR. The ZIKV viral RNA levels were normalized against reference gene ribosomal protein gene S7 (RPS7) (*n* = 3). Error bars indicate SD. This is a representative of three independent experiments. The low infection efficiency of mutant virus in controls (Con.) was rescued upon addition of VC, almost to a level comparable to that of the WT virus, whereas infection efficiency of WT virus was further increased with vitamin C treatment. Both changes were statistically significant. (B) H_2_O_2_ levels in the midguts of mosquitoes 24 h postinfection by blood feeding as described above. The basal H_2_O_2_ level (T156I) in the midgut of mutant virus-infected mosquitoes was significantly higher than that of the WT-infected mosquitoes. However, upon VC treatment, the H_2_O_2_ level in the former group (T156I+VC) was significantly reduced. The control (Con.) group was fed with only blood (*n* = 3). Error bars indicate SD. The *P* value was determined by unpaired *t* test. This is a representative of three independent experiments. (C) Schematic diagram of genes related to the reactive oxygen species pathway. The HPX family genes are involved in decomposition of H_2_O_2_, while the CuSOD_2_, DUOX1, and DUOX2 genes participate in synthesis of H_2_O_2_. NADPH oxidase (NOX), dual oxidase (DUOX), copper superoxide dismutase (CuSOD_2_), thiol peroxidase (TPX), and heme peroxidases (HPX). (D) Regulation of ROS pathway genes during ZIKV infection. Total RNA pooled from 20 mosquito midguts was extracted 24 h postinfection, and the mRNA levels of ROS-related genes were quantified by real-time PCR. Transcript levels of ROS degradation enzymes HPX8B and HPX8C were upregulated in WT-infected mosquitoes much higher than the mutant virus (T156I)-infected ones compared to the control (Con.) mosquitoes, which were fed with blood only. No significant change was detected in mRNA levels of ROS-generating enzymes upon viral infection (*n* = 3). Error bars indicate SD. This is a representative of three independent experiments.

We further investigated the mechanism by which the N-glycosylation of E protein facilitates invasion of the mosquito midgut by flaviviruses. It has been reported that the ROS pathway is heavily involved in the mosquito immune defense against midgut invasion by DENV (59). We speculated that N-glycosylation of the viral envelope might play a role in decreasing the ROS production, allowing successful infection of the mosquito midgut by ZIKV. To test this hypothesis, we utilized vitamin C, which has been previously demonstrated to inhibit ROS production (50, 59, 60). Specifically, vitamin C was added to the virus-containing blood meal during blood feeding, and viral RNA levels in the midgut were measured by real-time PCR. Consistent with a previous report on DENV that vitamin C can increase the mosquito infection rate by 3 times (59), ZIKV WT infection was increased by about 0.7 log fold after vitamin C treatment (Fig. 5A). Interestingly, the mutant virus infection, initially much lower than the WT, was also increased by about 3 log fold with vitamin C treatment and reached a level comparable to that in the WT infection (Fig. 5A). Therefore, vitamin C could rescue the midgut invasion by E N-glycosylation-negative ZIKV.

To further confirm the relationship between ROS and ZIKV infection, the ROS levels in the mosquito midguts were tested 24 h post-virus infection administered through blood feeding. The ROS level was downregulated ~10% by the WT virus compared to the control fed blood only and was further reduced by 20% with the addition of vitamin C. However, the ROS level in the mutant-infected midgut was 1.4 times that of the WT, whereas it was decreased by 44% with addition of vitamin C (Fig. 5B). Furthermore, the mRNA levels of genes related to ROS production and degradation were assayed by real-time PCR 24 h postinfection (Fig. 5C). The mRNA levels of ROS-producing enzyme, such as CuSOD_2_, dual oxidase 1 (DUOX1), and DUOX2, did not show any significant change after infection either by WT or the mutant. However, WT virus infection led to upregulation of the mRNA levels of ROS degradation enzymes HPX8B and HPX8C in the midgut by more than 200- and 400-fold, respectively, compared to the control group fed with virus-free blood (Fig. 5D). We speculate that this increased expression of ROS degradation enzymes in WT-infected mosquito midguts is responsible for the observed decrease in ROS levels. On the other hand, no such increase in the expression of ROS degradation enzymes was observed in mutant virus-infected mosquito midgut (Fig. 5D). Taken together, these results suggest a model according to which the N-glycosylation of the WT ZIKV E protein leads to a decrease in the ROS level via upregulation of genes related to ROS degradation, thereby facilitating viral invasion of the vector midgut.

## DISCUSSION

The N^153^- or N^154^-glycosylation on E protein is conserved among most vector-borne flaviviruses and is strikingly visible on the virus envelope under cryo-electron microscope (27). However, the glycosylation site is often lost *in vitro* during multiple passages in the mosquito or mammalian cell culture (16, 34, 35). In this work, using the ZIKV prototype MR766 strain and a clinically isolated Natal-RGN strain, we found that this glycan modification on E protein of the viral envelope is critical for vector midgut invasion by the virus. However, there was no significant effect on efficacy of viral infection or replication in either mammalian or mosquito cell culture systems. Our results suggest a high selection pressure for retention of E protein glycosylation of flaviviruses during vector transmission, as opposed to *in vitro* cell cultures that lead to the loss of E glycosylation of ZIKV laboratory isolates.

Mosquitoes are covered with a tough chitinous exoskeleton that provides protection against invading pathogens. However, their obligated blood feeding makes midgut the most accessible and susceptible tissue for viral infection. Although, mosquitoes lack the acquired immune system of mammals, during millions of years combating and coevolving with pathogens, the mosquitoes have adopted an efficient innate immune system, including such elements as RNA interference (RNAi), ROS production and antimicrobial peptides (50, 51). Concomitantly, certain strategies have evolved to enable the virus to cope with the immune system of mosquitoes. Here, we found that the N-glycosylation site on the viral E protein is a critical determinant of ZIKV efficiency for crossing the vector midgut barrier. We observed that upon removal of the E protein N-glycosylation site at position N^153/154^, midgut infection by both African and Asian lineages of mutant ZIKV viruses was dramatically decreased to almost undetectable levels, indicating a loss of invasive capacity when the virus is administered through the oral route. However, when administered intrathoracically by microinjection, bypassing the necessity of midgut-mediated uptake, the virus replicated to a level comparable to that of WT strains, indicating successful infection. Surprisingly, the observed infection of the midgut by the mutant virus when injected intrathoracically suggests that the midgut is still susceptible and permissive to the mutant virus. In order to cross the midgut barrier, flaviviruses need to suppress the vector immune defense system. Among the various immune pathways, ROS has been previously shown to play a key role in anti-DENV immune response (50, 59). Interestingly, the failure of midgut invasion by the E glycosylation-negative ZIKV could be rescued by vitamin C, an ROS inhibitor, indicating that glycosylated viral E protein suppresses ROS production in the mosquitoes, thus aiding the infection process. The change in ROS level in the midgut and alterations in the mRNA levels of ROS-related genes during viral infection provide further support to this hypothesis.

However, in contrast to our findings, YFV, which is a highly virulent mosquito-transmitted flavivirus, lacks the E N-glycosylation on the envelope (31). Earlier reports have demonstrated that secreted DENV and ZIKV viral NS1 proteins in human serum enhance the viral infection of mosquito midgut by antagonizing the ROS pathway (59, 61). Interestingly, the enhancement activity of NS1 is highly variable among different ZIKV strains due to the difference in the levels of NS1 in the sera (61). Although the NS1 protein in the human serum is not indispensable for the invasion of midgut by ZIKV and DENV, it is possible that the YFV could cope with the vector defense by NS1 or together with some other unidentified strategies.

Extensive research of the vector and host suggests that the N-glycosylations on the viral envelope interact with C-type lectins (29, 62–64). Glycosylation of envelope protein was bound by C-type lectins (CTLs), which facilitated ZIKV invasion by suppressing the downstream immune signaling pathways. However, loss of N-glycosylation abolished the interaction between CTLs and E envelope protein. Furthermore, it activated the Toll pathway, leading to the burst of ROS. The oxidative stress blocked the transmission of ZIKV through mosquito midgut.

Mosquito-borne flaviviruses can persistently infect their vectors, causing no obvious pathogenicity due to low viral replication levels, and the vector immune system serves as the limiting factor. An antiviral factor, AaHig, has been identified to control DENV and JEV replication in the nervous system of *A. aegypti*. Knockdown of AaHig enhances the viral replication in the vector and reduces the life span of mosquitoes, while overexpression of AaHig eliminates viral replication in the nervous system (65). *Wolbachia* is a promising transmission-blocking agent that can block DENV infection in the vector and was recently found to function by increasing the ROS levels in the midgut (50). Our data suggest the N^154^-glycosylation on the E protein blocks the ROS pathway and is critical for the vector competence of ZIKV. Further investigation of the interactions between arboviruses and their vectors will facilitate development of more powerful transmission-blocking strategies for arboviral control.

## MATERIALS AND METHODS

### Ethics statement.

All animal and mosquito experiments were performed strictly following the principles of bioethics and were subjected to supervision by the Bioethics Committee of the Institute of Zoology, Chinese Academy of Science. ZIKV experiments were performed under biosafety level 2 (BSL2) and animal BSL3 (A-BSL3) containment.

### Cells and antibodies.

HEK293T, Vero, and Huh7.5 cells were maintained in DMEM plus 10% fetal bovine serum (FBS) and l-glutamine at 37°C with 5% CO_2_. C6/36 cells were maintained in RPMI 1640 plus 10% heat-inactivated FBS and l-glutamine at 28°C with 5% CO_2_. 4G2 is a mouse monoclonal antibody (MAb) that recognizes the fusion peptide of E protein of all flaviviruses, including DENV and ZIKV (66, 67).

### *De novo* synthesis of ZIKV infectious clones and mutagenesis.

The complete sequences of the ZIKV MR766 strain and Natal-RGN strain were obtained from GenBank. The MR766 strain was synthesized using LC002520 (GenBank sequence accession number) as the template, except for the E N^154^ region, which was obtained from HQ234498. The whole genome was divided into three overlapping segments, including A (positions 1 to 2596), B (positions 2577 to 7076), and C (positions 7057 to 10807), and synthesized by Genewiz Suzhou. All of the segments were further assembled into low-copy-number vector PACYC177 with a T7 promoter in front of the 5′ terminus and a NotI restriction site following the 3′ end using NEBuilder Hi-Fi DNA assembly master mix (NEB Biolabs).

The Natal-RGN strain was synthesized using sequence KU527068 as the template and divided into three pieces, including A (positions 1 to 3370), B (positions 3325 to 7680), and C (positions 7640 to 10808). To reduce the toxicity of the genomic DNA, an intron was inserted after nucleotide 2711 of the viral sequence. All three pieces were assembled into low-copy-number vector PACYC177 with a CMV promoter in front of the 5′ end and a hepatitis delta virus (HDV) ribozyme (RBZ) terminal site following the 3′ end.

*E. coli* strain TOP10 was used for all the transformations and cultured at 30°C. The final infectious cDNA clones of the ZIKV MR766 and ZIKV Natal-RGN strains were fully sequenced to ensure the absence of any mutation. E-T156I (AC^1442^A→AT^1442^A) mutation was introduced into the MR766 infectious clone by site-directed mutagenesis. ZIKV Natal-RGN E-T156I was generated by mutating AC^1443^A^1444^ to AT^1443^C^1444^.

### Rescue of infectious clones.

ZIKV MR766 was rescued as described previously (54). Briefly, viral RNAs were *in vitro* transcribed and electroporated into HEK293T cells by a Gene Pulser apparatus (Bio-Rad) in 4-mm cuvettes with settings of 300 V and 15 ms. Viruses in the supernatant were collected 3 to 4 days postelectroporation and frozen at −80°C as stocks.

ZIKV Natal-RGN was rescued by transfection of the infectious clone plasmid into HEK293T cells with calcium phosphate. After 6 to 7 days, cultured cells were collected and stored at −80°C.

### ZIKV RVPs.

The flavivirus RVP system was a gift from Ted Pierson (53). Codon-optimized capsid-prME of ZIKV MR766 strain was synthesized by Genewiz and subcloned into the pCDNA3.1 vector (designated pZIKV-MR766-CprME). For RVP production, 12 µg of pZIKV-MR766-CprME and 3 µg of the replicon plasmid pWNVII-Rep-GFPZeo were cotransfected into HEK293T cells by calcium phosphate. At 12 h posttransfection, the medium was changed to low-glucose (1 g/liter) DMEM supplemented with 7% FBS at 28°C. RVPs in the supernatant were harvested at 72 h posttransfection and titrated on Huh7.5 cells by a focus-forming assay. E proteins in the cell lysate were subjected to immunoblotting with 4G2 antibody.

### Plaque assay.

Vero cells were plated in 24-well plates 24 h before infection. At 200 µl per well, 10-fold serial dilutions of virus samples in DMEM (supplemented with 2% FBS and 1% penicillin/streptomycin) were added to cells for 2 h at 37°C and then replaced with 500 µl of MEM containing 1.2% methylcellulose plus 5% FBS. Plates were incubated at 37°C with 5% CO_2_ for 5 days and then fixed for 15 min with 4% paraformaldehyde and stained with 1% crystal violet solution.

### Endoglycosidase analyses of E protein.

Viral particles in the supernatant were pelleted by ultracentrifugation and then treated with PNGase F (NEB) overnight under nondenaturing reaction conditions at room temperature. Enzyme-treated samples were analyzed by Western blotting under a nonreducing condition using anti-E 4G2 antibody.

### Mosquito infection experiment.

*A. aegypti* (UGAL/Rockefeller strain) mosquitoes were maintained in the laboratory as described previously (68). The adults were fed on water and 10% (wt/vol) sugar solution. Five- to 6-day-old female mosquitoes were used for this study.

For oral infection, 5- to 6-day-old females were starved for about 24 h before ZIKV oral infection. Viruses were diluted to 1.5 × 10^5^ PFU/ml in 50% mouse blood and 50% DMEM and preheated at 37°C for 30 min before feeding. Then, mosquitoes were allowed to suck the virus mixtures at 37°C through a very thin Parafilm for 30 min. Subsequently the mosquitoes were chilled at 4°C for 10 min, and those full of blood were picked up for further experiments. For the vitamin C (VC) rescue experiments, VC was added to the blood-virus mixture at a final concentration of 20 mM.

For intrathoracic microinjection, 5- to 6-day-old females were intrathoracically injected with 60 PFU of virus diluted in 200 nl DMEM. Subsequently, mosquitoes were fed with water and 10% sugar solution.

### RNA extraction and real-time PCR analysis.

Total RNA samples from pooled tissues or whole mosquitoes were extracted with Trizol (Invitrogen). Total RNA from a single mosquito was extracted by the TGuide cell/tissue/plant RNA kit (Tiangen). Quantitative PCR was performed using a One Step SYBR PrimerScript reverse transcription (RT)-PCR kit (TaKaRa) on Applied Biosystems QuantStudio. The ZIKV viral RNA levels were normalized against reference gene ribosomal protein gene S7 (RPS7). The sequences of the RPS7 primers were as follows: sense, 5′-TCAGTGTACAAGAAGCTGACCGGA; antisense, 5′-TTCCGCGCGCGCTCACTTATTAGATT. The sequences of the ZIKV MR766 primers were as follows: sense, 5′-GGGGAAACGGTTGTGGACTT; antisense, 5′-CTGGGAGCCATGCACTGATA. The following amplification program was used: reverse transcription at 42°C for 5 min with incubation at 95°C for 10 s, followed by 40 cycles of 95°C for 5 s and 60°C for 20 s. Information collection and melt curve analysis were done following the instrument’s operation manual.

### Immunofluorescence assay.

Virus-infected mosquitoes were chilled at 4°C for 10 min, and the midguts were dissected in PBS. After being washed 3 times with PBS, midguts were fixed with 4% paraformaldehyde (PFA) at room temperature for 30 min and permeabilized with 0.5% Triton X-100 at room temperature for 10 min. Subsequently, tissues were blocked with 3% bovine serum albumin (BSA) in phosphate-buffered saline (PBS) at room temperature for 1 h. To detect ZIKV E protein, tissues were incubated with mouse MAb 4G2 overnight at 4°C. After being washed with PBS 6 times, tissues were incubated with Alexa Fluor 488 goat anti-mouse secondary antibody (1:400) for 1 h at room temperature. Hoechst 33342 was added at 1 μg/ml to stain the nucleus. The resulting fluorescence was detected by confocal microscopy (Zeiss LSM 710; Germany).
