# Supplementary figures and images for: N-glycosylation of Viral E Protein Is the Determinant for Vector Midgut Invasion by Flaviviruses
Source: mBio. 2018 Feb 20;9(1):e00046-18. doi: 10.1128/mBio.00046-18 (PMC5821097; doi:10.1128/mBio.00046-18)

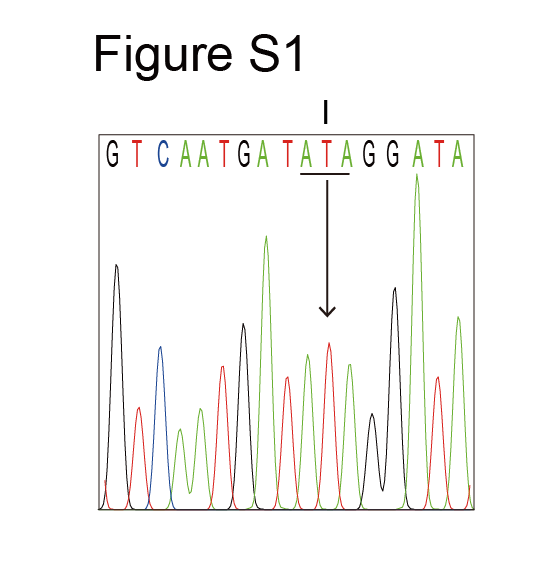

Supplement: FIG S1 [file mbo001183727sf1.tif]

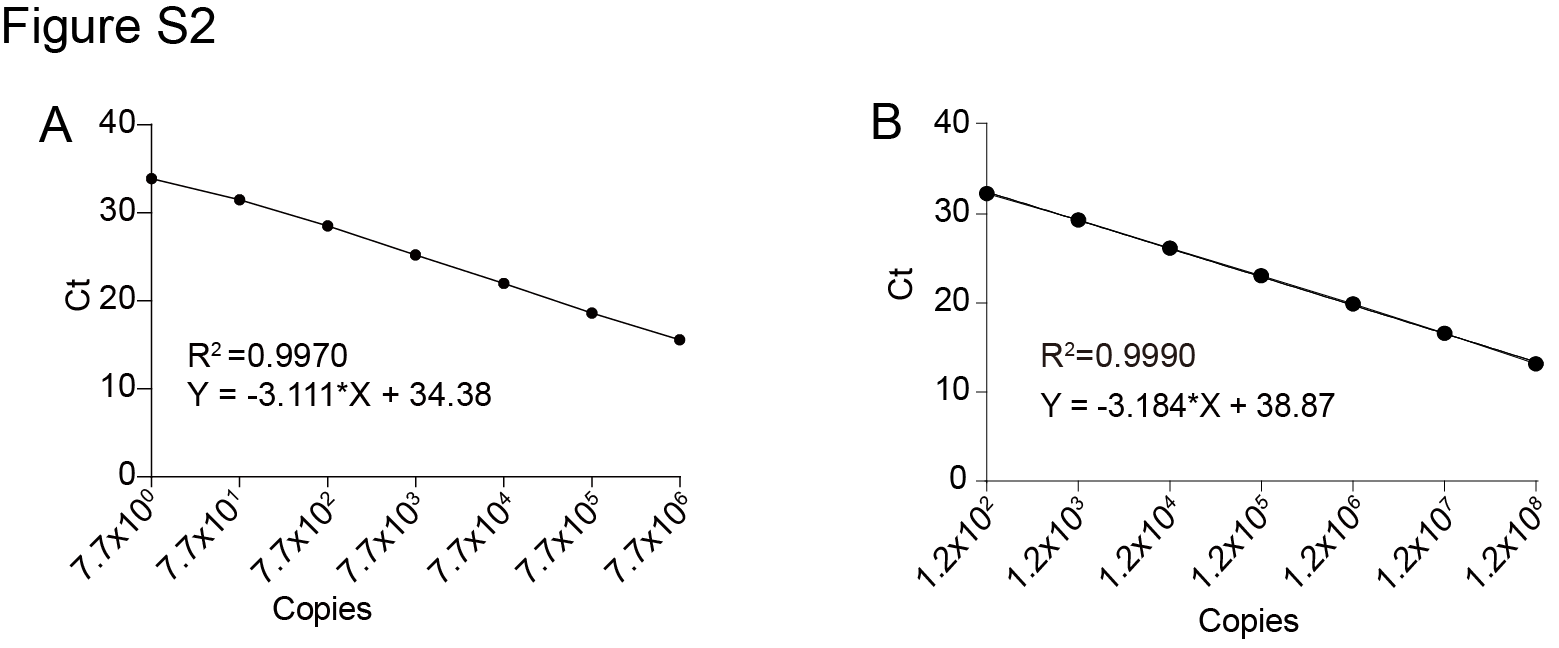

Supplement: FIG S2 [file mbo001183727sf2.tif]

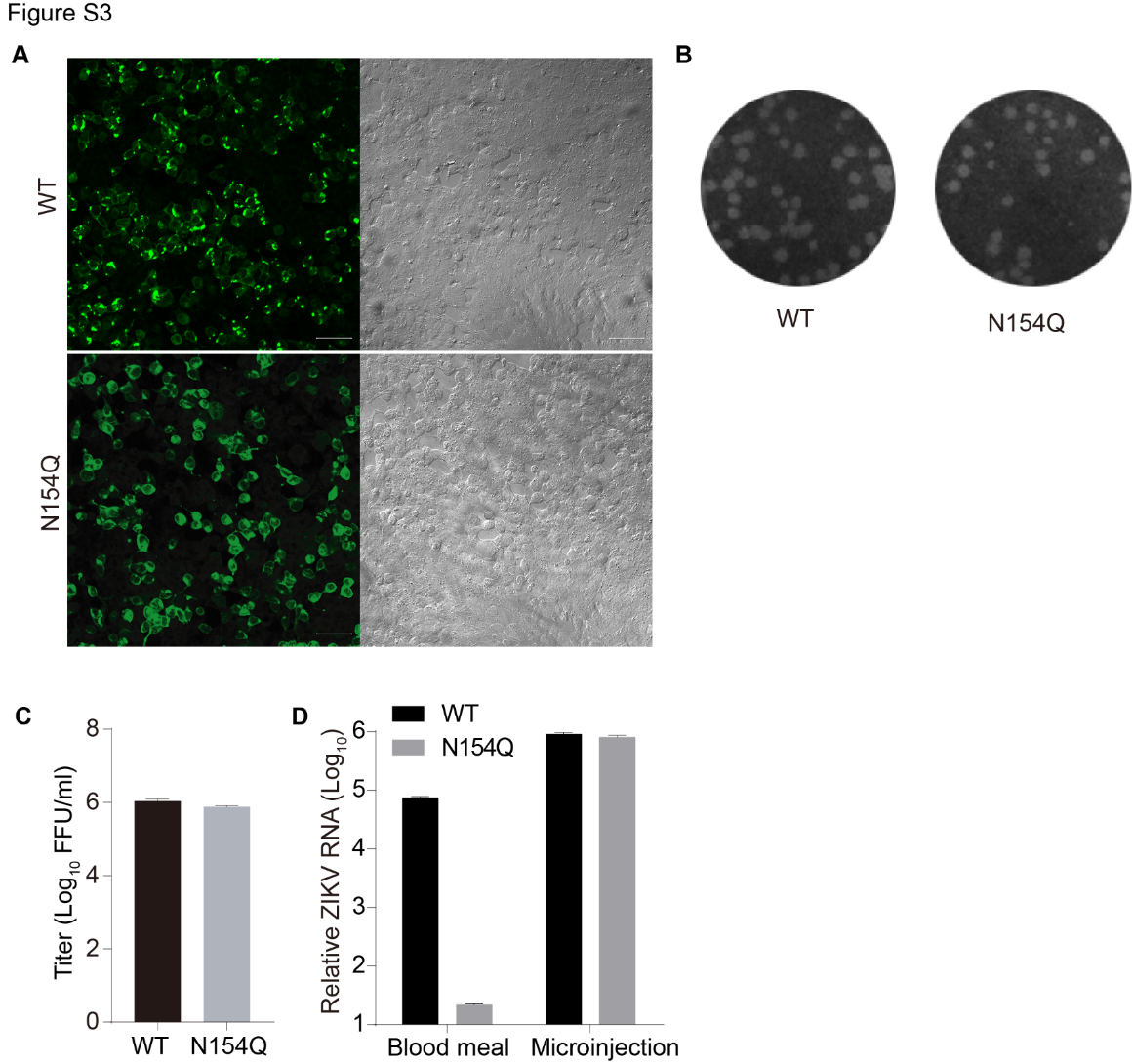

Supplement: FIG S3 [file mbo001183727sf3.tif]
